# Characteristics of Selected Somatic and Motor Abilities of Youth Soccer Players with Diabetes Type 1 Treated with Insulin Pump Therapy

**DOI:** 10.3390/ijerph18073493

**Published:** 2021-03-27

**Authors:** Magdalena Krzykała, Katarzyna Domaszewska, Małgorzata Woźniewicz-Dobrzyńska, Jakub Kryściak, Agata Konarska, Aleksandra Araszkiewicz, Dorota Zozulińska-Ziółkiewicz, Andrzej Gawrecki, Grzegorz Biegański, Jan M. Konarski

**Affiliations:** 1 Department of Recreation, Poznań University of Physical Education, 61-871 Poznań, Poland; dobrzynska@awf.poznan.pl; 2 Department of Physiology and Biochemistry, Poznań University of Physical Education, 61-871 Poznań, Poland; domaszewska@awf.poznan.pl (K.D.); j.krysciak@awf.poznan.pl (J.K.); 3 Department of Physiotherapy, Stanisław Staszic University of Applied Science, 64-920 Piła, Poland; konarskaag@gmail.com; 4 Department of Internal Medicine and Diabetology, Poznan University of Medical Sciences, 61-701 Poznań, Poland; olaaraszkiewicz@interia.pl (A.A.); zozula@box43.pl (D.Z.-Z.); pompainsulinowa@wp.pl (A.G.); 5 Department of Infectious Diseases and Child Neurology, Poznan University of Medical Sciences, 61-701 Poznań, Poland; grzbieg@mp.pl; 6 Theory of Sport Department, Poznań University of Physical Education, 61-871 Poznań, Poland; konarski@awf.poznan.pl

**Keywords:** children, physical fitness, type 1 diabetes

## Abstract

Long-term insulin treatment can slow the growth process and decrease physical fitness level in children. In diabetic children, these two developments should be constantly monitored. The aim of the present study was to examine differences in somatic and physical fitness characteristics between soccer-training boys with type 1 diabetes and healthy boys of the same age (reference values based on Polish population norms for somatic and motor parameters). The participants were 94 boys (8–17 years), diagnosed with diabetes, who participated in soccer training on a regular basis and received routine medical care. The study involved (a) anthropometric and body composition measurements, (b) general motor ability assessments, and (c) comparison of those characteristics with the healthy Polish population. The diabetic boys were found to have lower levels of almost all somatic traits and motor abilities as compared with the healthy boys (*p* ≤ 0.05). Handgrip strength was a variable with the smallest difference between the two groups. The observed differences indicate the necessity to design an appropriate control and assessment system based on simple medical and fitness field tests for diabetic children and adolescents. It will allow optimizing advanced training as well as minimize health risks before, during, or after exercise.

## 1. Introduction

Type 1 diabetes (T1D) is a prevalent autoimmune chronic disease caused by the destruction of pancreatic beta cells, which leads to insulin deficiency. It has well-known short- and long-term implications [1], such as impaired linear growth at prepubertal and pubertal age [2], or even severe impairment of growth and development known as Mauriac syndrome [3]. T1D accounts for 5–10% of the total cases of diabetes, and its prevalence has been steadily increasing in children under 15 years [4]. Once diagnosed this complex disease requires significant lifestyle changes, which may be difficult for children because of differences in growth and developmental levels. Diabetes management not only involves self-care, but also proper understanding of the impact of diabetes on the child, their activities in daily living, and acceptance of the disease [5].

Physical activity is an integral part of people’s lives. The main challenges for physically active people with T1D are hypoglycemia, fear of losing diabetes control, and insufficient knowledge about their behavior during physical activity [6]. However, thanks to the application of new technologies in diabetes treatment, these barriers have been largely overcome in the last decade [7]. The use of insulin analogs, insulin pumps, and continuous glucose monitoring (CGM) systems has made physical activity, including school, recreational, and professional sports, more common among diabetics [8]. People with T1D can now practice all types of sports, including those requiring maximum physical performance and extreme sports [9]. Teenagers with diabetes can now find information on how to prepare for participation in different forms of physical activity and sports, both amateur and professional [9,10,11,12,13].

A review of the latest reports from clinical trials in children with T1D points to the need for research related to their fitness and somatic characteristics compared with healthy populations [14,15]. The most important question is how an extended period of insulin treatment affects diabetic children [14,15]. Despite the immense significance of this issue, especially regarding children and adolescents with T1D, there has been no research examining the differences between children with and without T1D. The purpose of this study was to evaluate differences in somatic and physical fitness characteristics of boys with T1D, who took part in the First Polish Soccer Championships for Children and Adolescents with Diabetes, and compare the measurements of these characteristics with norms for a healthy Polish population in the same age range.

## 2. Materials and Methods

In April 2017, Diabetes Poland organized the first “Polish Soccer Championships for Children and Adolescents with Diabetes” in Gniezno, during which the GoalDiab study was conducted, involving measurements of a number of participants’ anthropometric and physical fitness characteristics. The study sample consisted of diabetic boys aged 8–17, who regularly used insulin pumps (continuous subcutaneous insulin infusion—CSII). The study was conducted in accordance with the Declaration of Helsinki, and the study protocol was approved by the Human Ethics Research Committee of the Karol Marcinkowski Medical University in Poznań (Poland). Parents/legal guardians signed their approval for their children’s participation in the research.

### 2.1. Subjects

The study group included 155 boys with diagnosed T1D who participated in regular soccer (football) training and received routine medical care in 11 diabetes care centers in Poland. In all, 94 boys were qualified for the analysis. One of the key inclusion criteria was the use of insulin pumps by participants. Capillary blood samples were taken for HbA1c assessment (D-10 Hemoglobin A1c Program (Bio-Rad Laboratories, Hercules, CA; Bio-Rad, Marnes-la-Coquette, France)) on the day before the tournament and on the first day of the tournament.

Inclusion criteria were T1D, male sex, age 8–17 years, intensive insulin therapy using CSII, lack of other significant comorbidities being a contraindication for physical activity, diabetologist consent and qualification, and signed informed consent of the parent or legal guardian and the adolescent.

Exclusion criteria were advanced chronic complications of diabetes; severe non-proliferative or proliferative retinopathy; diabetic maculopathy; diabetic neuropathy; diabetic nephropathy (stage III–V chronic kidney disease); diabetic ketoacidosis or severe hypoglycemia in the last 30 days; already identified relevant cardiovascular, respiratory, or orthopedic diseases; and lack of signed consent by parents or legal guardians for participation in the tournament and in the study. Individuals using insulin pens (multiple daily injections—MDI) were excluded from the analysis.

### 2.2. Study Protocol

Before the tournament, the participants with their parents or legal guardians completed a questionnaire about the duration of diabetes, treatment method, comorbidities, history of acute and chronic complications, and medication use. The filled questionnaires were analyzed by diabetologists and sports medicine physicians.

#### 2.2.1. Assessment of Anthropometric Parameters

To divide the boys into chronological age subgroups, the whole year as the mid-point of the range was given, e.g., 12.50 to 13.49 = 13.0 years; 13.50 to 14.49 = 14.0 years. To assess body mass index (BMI) using Cole’s cutoff points, half-year ranges were also used, e.g., 13 years = 12.75 to 13.24 years; 13.5 years = 13.25 to 13.75 years; 14 years = 13.75 to 14.24 years. To assess BMI in relation to individual age, two sets of cutoff reference tables were used for the equivalent of BMI: 16, 17, and 18.5 kg/m^2^ (thinness), and 25 and 30 kg/m^2^ (overweight and obese) at the age of 18 [16,17,18]. This index is recommended for children of different ages between 2 and 18 years. To compare differences between the healthy population norms and the participants with T1D in body height and body weight, as well as the waist and hip circumference, the reference data from Kułaga et al. [19] and Świąder-Leśniak et al. [20] were used, respectively.

The study protocol included anthropometry and body composition assessments. Physical measurements were taken by a highly trained technician in accordance with the International Society for the Advancement of Kinanthropometry recommendations [21]. Standing height was measured to the nearest millimeter using a stadiometer (GPM, Zurich, Switzerland). To assess body fat distribution, waist circumference (WC) was measured. Participants were in a standing position at the end of normal expiration, measured in the horizontal plane at the level of the narrowest point between the lower costal border and the iliac crest, to the nearest 0.2 cm, using a non-stretchable tape. Hip circumference (HC) was measured at the level of the greatest protrusion of the gluteal (buttock) muscles. Fat distribution was assessed using the waist-to-hip ratio (WHR) and calculated by dividing WC by HC. Waist-to-height ratio (WHtR) was calculated as the ratio of WC to body height to predict the risk of cardio-metabolic complications of obesity. This index, as well as the WC, are recommended as part of anthropometric measurements to identify and monitor obesity in children [22,23]. A WHtR cutoff of 0.5 can be used in different sex and ethnic groups and is generally accepted as a universal cutoff for central obesity in children (aged ≥6 years) and in adults.

Body weight and body composition (fat mass—FAT, fat free mass—FFM, total body water—TBW) of diabetic children were estimated with bioelectric impedance analysis using a Tanita MC-780 MA analyzer (Tanita Corp., Tokyo, Japan), in accordance with the manufacturer’s guidelines. Participants were instructed to refrain from exercising, eating or drinking anything other than water for 3 h before testing, and to void their bladder to ensure that test results were not influenced by body temperature, breathing rate, or presence of food or beverage in the gastrointestinal tract [24]. This method is commonly used in field surveys and as a supplement to conventional anthropometry [25].

#### 2.2.2. Assessment of General Motor Abilities

Four functional tests were administered in the following order: the Ruffier test (endurance index, points), handgrip strength of the right and the left hand (declared dominant hand; general strength level, kg), and 5 m dash (speed, s).

### 2.3. Endurance (Ruffier Index)

The level of physical fitness was determined using the Ruffier test for children on the day before the tournament. The Ruffier test was preceded by a 5 min rest in a sitting position. Then, the measurement of heart rate (HR) was conducted within 15 s (P1), and again after performing 30 squats in 45 s (P2). The third HR measurement (P3) was made within 15 s of the first minute of restitution. Then, using the formula IR = (4 (P1 + P2 + P3) − 200)/10, the Ruffier test index (RI) was calculated. The following values were used to interpret the RI: RI = 0 = very good, 0 < RI < 5 = good, 5 < RI < 10 = moderate, 10 < RI < 15 = poor, RI > 15 = very bad [26]. A Polar Team 2 unit (Polar Electro Oy, Kempele, Finland) and Polar Team software were used for HR recording.

### 2.4. Handgrip Strength

Handgrip strength was measured using a hand dynamometer (Lafayette model 78010, Lafayette Instrument Company, Lafayette, IN, USA). Each participant adopted an erect posture with arms at their side, not touching their body, and keeping the elbow bent slightly during measurement [27]. The testing range on a dual scale was 0–100 kg. The test was repeated three times with a pause of 1 min between each trial to avoid the effects of muscle fatigue. The best trial was recorded and presented in kg. Both the right and the left hand were tested in each participant and, the highest grip strength value of the hand declared as dominant was used for detailed analysis.

### 2.5. Running Speed (5 m dash)

Each participant ran a distance of 5 m from a standing start 0.5 m behind the starting line. The time from crossing the starting line to crossing the end line was measured to the nearest 0.001 s using a digital laser photocell system (Witty, Microgate, Italy). The run was repeated twice. The better time was converted to velocity (m·s^−1^) for the analysis [28,29]. The reference values used to assess speed were the norms for 5 m dash by Zając and Waśkiewicz [30].

### 2.6. Data Analysis

The data were compiled using standard statistical methods. The Shapiro–Wilk test was used to check the normality of distribution. Basic descriptive characteristics were calculated as arithmetic means (M) and standard deviation (SD). To compare the somatic variables between the diabetic players and the reference values for the healthy population a single sample t-test was used. The level of statistical significance was set at *p* < 0.05, *p* < 0.01 and *p* < 0.001; with *p* < 0.1 indicating a tendency towards significance; and *p* ≥ 0.05—lack of statistical significance. The differences between the reference values and the measurement results were assessed by means of a significance test of the differences for two mean values. Quantitative parameters were presented using means and percentages. The measurement results of the Polish boys aged 8–17 with T1D for fitness, body height, body weight, and waist and hip circumference compared with the healthy population [19,20] were assessed using the following standards: the Ruffier test results—reference data of Faik et al. [26], 5 m dash results—reference data for youth soccer players by Zając and Waśkiewicz [30], and handgrip results—the ranges indicated by Dobosz for Polish children [31]. The Body Mass Index (BMI) was calculated to classify all youth soccer T1D players, using sex-specific IOTF (International Obesity Task Force) cut-offs, according to Cole et al. [16,17]. All statistical analyses were performed with the use of Statistica version 13.3 (TIBCO Software Inc., Palo Alto, CA, USA, 2017).

## 3. Results

The data on the somatic and motor abilities of 94 boys with T1D are presented in Table 1.

### 3.1. Anthropometric Parameters

**Figure 1:**
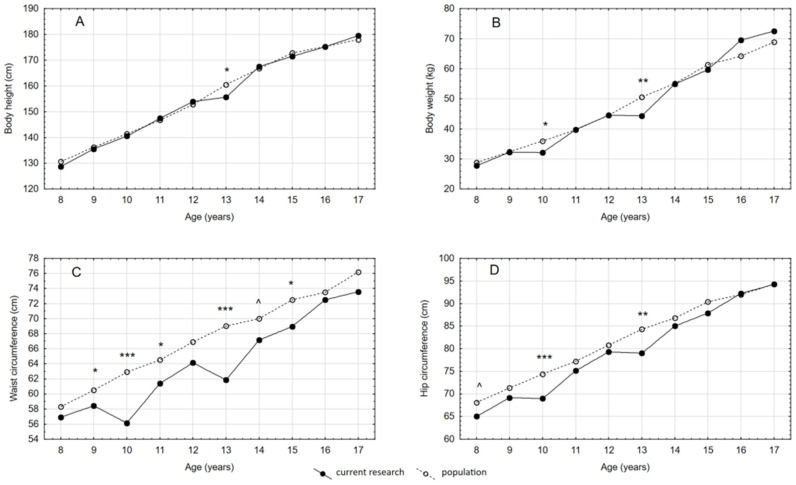
Measurement results of Polish boys aged 8–17 years with type 1 diabetes for (**A**) height, (**B**) weight, (**C**) waist, and (**D**) hip circumference compared with the healthy population norms [18,19]. * *p* < 0.05, ** *p* < 0.01, *** *p* < 0.001—statistically significant; ^ *p* < 0.1—tendency towards significance; *p* ≥ 0.05—statistically non-significant.

Means and standard deviations of all traits were calculated for all age categories. Body height and body weight increased with age. The analysis showed that, in the case of body height, a significant difference between the groups was noted only in 13-year-old boys (*p* = 0.0344) (Figure 1A). In terms of body weight, a significant difference was found in boys aged 10 and 13 (*p* = 0.0302 and *p* = 0.0050, respectively), but it was generally fluctuating (Figure 1B). Mean values for WC and HC also increased with age. The results indicated that in all age groups the diabetic boys had lower values of those characteristics than the reference values for the healthy population (Figure 1C,D) [18,19].

Significant differences in waist circumference were found in boys aged 9 (*p* = 0.0297), 10 (*p* = < 0.0001), 11 (*p* = 0.0199), 13 (*p* = < 0.0001), and 15 (*p* = 0.0344) years, and a tendency towards significance in boys aged 14 years (*p* = 0.0643). In hip circumference, a significant decrease was noted in diabetic boys aged 10 and 13 years (*p* = 0.0006 and *p* = 0.0030, respectively), and a tendency towards significance in diabetic boys aged 8 years (*p* = 0.0682) as compared with the healthy population.

Because the increase was relatively higher for HC than for WC, the mean WHR decreased for boys from 0.87 to 0.78 (Table 1). WHR values for all participants were below 1, which means that they had gynoid fat distribution with accumulated fat tissue in the lower body, especially the buttocks, thighs, and hips. WHtR changed relatively little with age and was under 50%.

Percent of FAT was generally stable for boys aged 8–12 years, and decreased at the age of 13 in the study group. Conversely, %FFM and %TBW showed an increasing trend for boys aged 13–17 years (Table 1).

The BMI analysis, according to Cole’s cut-offs for thinness, overweight, and obesity showed that among the 94 participants, 4 were thin, 1 was mid-thin, 3 were moderately thin, 10 were overweight, and 80 were of normal body weight (Table 2).

### 3.2. Motor Abilities

**Figure 2:**
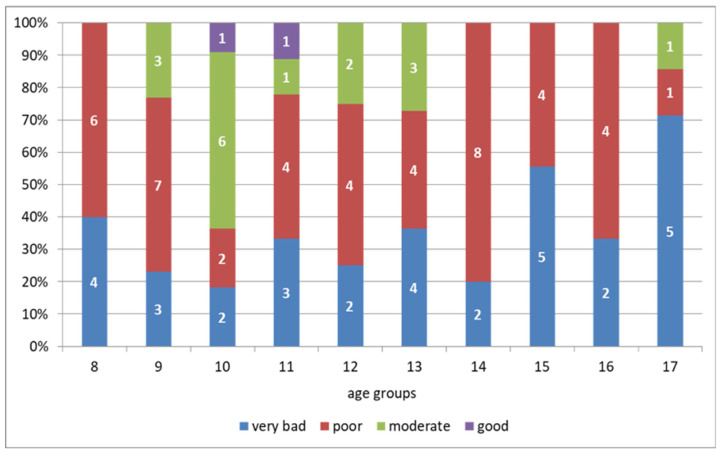
Ruffier test results according to reference data of Polish children aged 8–17 years with type 1 diabetes [25].

The mean RI score for all participants was 13.6 ± 4.0 points, with individual and intergroup differences. Among the examined children, around 2% had good circulatory efficiency, 17%—moderate, and 47% and 34%—poor or very bad circulatory efficiency, respectively. Figure 2 shows the number of participants with particular performance levels in each age group.

The analysis of the 5 m dash results by age group revealed that only the 9 and 10 year-old boys were classified as average compared with the norm for youth soccer players [30]. The other age groups did not reach even the average level of sprint performance premised for youth athletes (Table 3). Therefore, it can be said that the diabetic children and adolescents attained generally poorer results in 5 m dash compared to healthy population (71%). Significantly slower were the boys aged 13, 14, and 17 years. It is important to note that there are no reference values for 8 year-old children and it was not possible to compare their test results with the norms for the healthy population.

The analysis of grip strength of the dominant hand in T1D children showed that most of the boys, i.e., 63 of 94 (67%), scored within the reference range for the Polish population [31]. Scores below the norm were noted in 24 (26%) and above the norm in 7 (7%) out of the studied 94 youth soccer T1D players (Table 3).

## 4. Discussion

The main aim of the study was to evaluate the somatic characteristics and simple field-tested general motor abilities of boys with T1D. In general, the growth variables appear to be significant indications for metabolic control in T1D and the child’s overall health. This is especially relevant for sport-training children and adolescents with T1D [32]. Rohrer et al. found that a critical phase for growth gain is puberty, given related hormonal changes [33]. The earlier its onset, the longer and more severe the disease, as well as the greater the impact on growth and pubertal development [34]. During the course of the disease, growth deceleration has been reported in many countries, e.g., Austria, Brazil, the Czech Republic, and Germany [35,36,37]. Similar observations were made by other authors, who have stated that conventional therapy for diabetic children could be associated with impairment of physical growth and delay in pubertal development [38,39,40]. On the other hand, a few studies showed no effect of diabetes on growth [35,41,42].

Other authors indicate that growth retardation particularly concerns children with poor diabetes control as, for example, affirmed by body height [39,43]. Moreover, in the 21st century, lower body height is a potential long-term complication of poorly controlled T1D [32], thus optimal metabolic control and monitoring of somatic and fitness parameters in children and adolescents are crucial. In the present research, all participants were under good metabolic control, which probably explains the small differences between the diabetic children and the healthy population in body height and body weight.

WC is a good indicator of fat distribution around the waist, which is a risk factor and a predictor of non–insulin dependent diabetes mellitus [44]. WHtR has been used in research and clinical settings and is recommended as the preferred measure of central obesity in both sexes across age groups [45,46]. According to the literature, values of this index not exceeding 50% are considered free of weight-dependent health risks [47]. In the present study no children exceeded this index level.

During growth changes in body fat occur. The noted decreasing %FAT with age in diabetic boys from 13 years of age was consistent with similar observations among healthy children [48,49]. In the present study, lower %FAT was noted at the age of 15. It appears to reach a plateau or change only slightly near the time of the adolescent growth spurt for healthy boys, between 13 and 15 years, and reach its lowest point at 16 to 17 years, and then gradually increase into young adulthood [22]. This is related to %FFM changes at that time. Tuvemo [50] and Bartzs [51] indicated that weight gain in young diabetics was due to an increase not in fatty tissue but in muscle mass. Compared with the values in metabolically normal controls, the %FAT was lower for the diabetics than the controls. Generally, the achievement of correct treatment results affects the physical fitness of boys with T1D. In some studies, children and adolescents with diabetes displayed physical fitness levels comparable with those of healthy controls [52,53], although they can also be worse [54,55,56]. The Ruffier test results in the present study confirm it. In the studied group of diabetic boys, the RI ranged between 3.0 and 23.2 points with an average of 13.6 points. Most respondents (46%) had a poor level of physical fitness. This is comparable with the results of healthy children in earlier studies in Poland [26,57]. The unsatisfactory results of the Ruffier test cannot be explained only by the emotions associated with the test. From a physiological point of view, this test examines post-exercise HR restitution. On the other hand, the HR increase depends on the emotional state only at the beginning, and is later conditioned by autonomic and endocrine factors. It is probably caused by the slow post-exercise HR recovery. This mechanism has been found in individuals with reduced levels of physical fitness, cardiovascular disease, or high blood glucose concentration.

In the 5 m dash test, the diabetic boys ran much slower than their healthy peers of the same age. Sprint abilities are one of the most important elements of soccer players’ general preparation, and are connected with development by age [22,58]. Further research is necessary, in part because short-duration and intermittent exercise is more recommended than long-duration and high-intensity activity. Generally, children aged 5–17 years should engage in 60 min or more of moderate- to vigorous-intensity physical activity daily [59]. However, children with T1D should exercise with caution due to the risk of hypoglycemia (especially in the night after exercise; [60]) and ensure stable blood glucose levels before, during, and after exercise [61]. Maly et al. in their study of youth soccer players found that boys with T1D were slower in sprinting over the distance of 5 m [62].

Some basic anthropometric variables, such as body height, weight, and BMI have been reported to influence handgrip strength in children [63]. According to Sartorio et al., the increase in handgrip strength with age depends on the parallel increase in muscle mass [64]. In the present study, the higher %FFM, the higher handgrip strength was observed, but—especially in older children—this value was lower in comparison to the healthy population. According to Malina et al., handgrip strength (which reflects general fitness) increases most dynamically after the growth spurt that occurs after 14 years of age [22]. However, in the present study, in most age groups, the handgrip strength results were lower than the norm for the general population [31]. This could suggest that children with T1D are generally weaker their healthy peers. This was also confirmed by Hagag et al. [58]. Moreover, upper body muscular strength (measured with the handgrip strength test) was negatively associated with fasting insulin [11,65].

The present study has some limitations. One of them is data collection from a cross-sectional study, thus the obtained results and associations should be interpreted with caution. A longitudinal study would enable a more accurate analysis of the data and could reveal more reliable effects of T1D on the morphological and functional parameters in children undertaking regular physical activity. It did not take into account the participants’ biological maturity stage, and the analysis was based on the chronological age similar to the rules of official youth soccer competitions. Prospective research should focus on this particular aspect using invasive and/or non-invasive methods considering the lack of such data about T1D soccer players in sports literature. Although we did not include a control group, we compared the obtained results with normative values of a healthy population. The strengths of our study include a relatively homogenous sample of soccer-training boys with type 1 diabetes as well as the use of reliable field tests.

## 5. Conclusions

Type 1 diabetes might negatively affect the growth and development, as well as physical fitness, of children. The present study confirmed the importance of regular assessment of key somatic and fitness parameters in the context of growth of children with T1D in relation to the general, healthy population. Moreover, the found differences indicate the necessity to design an appropriate control and assessment system based on simple field tests, including both the medical and fitness areas of observation for this group of sport participants. This will allow optimizing training processes on different advancement levels as well as minimize health risks of T1D before, during, or after exercise.

## Figures and Tables

**Table 1:** Main characteristics of children with type 1 diabetes in all age categories (M ± SD).

Variable	Boys’ Age Groups									
	8	9	10	11	12	13	14	15	16	17
	(*n* = 10)	(*n* = 13)	(*n* = 11)	(*n* = 9)	(*n* = 8)	(*n* = 11)	(*n* = 10)	(*n* = 9)	(*n* = 6)	(*n* = 7)
Diabetes duration (years)	2.8 (1.53)	4.54 (2.44)	5.18 (2.52)	5.00 (3.20)	4.46 (3.74)	4.91 (3.11)	4.13 (2.69)	7.67 (4.09)	7.50 (5.05)	10.42 (5.41)
HbA1c (%)	7.15 (0.78)	7.3 (0.58)	7.3 (1.10)	7.3 (1.03)	7.5 (1.08)	7.6 (072)	8.3 (1.99)	7.6 (0.66)	8.2 (1.28)	8.0 (1.21)
Body height (cm)	128.7 (4.72)	135.5 (5.85)	140.5 (9.95)	147.4 (7.87)	153.9 (5.79)	155.6 (6.59)	167.5 (4.82)	171.5 (6.46)	175.2 (10.60)	179.5 (5.22)
Body weight (kg)	27.76 (4.79)	32.23 (5.29)	32.15 (5.06)	39.75 (6.78)	44.57 (7.31)	44.35 (5.79)	54.95 (6.28)	59.7 (9.42)	69.56 (12.67)	72.52 (7.51)
Waist circumference (cm)	56.9 (4.22)	58.42 (3.04)	56.13 (3.26)	61.38 (3.22)	64.12 (5.39)	61.86 (2.41)	67.15 (4.28)	68.94 (4.19)	72.5 (4.59)	73.57 (4.71)
Hip circumference (cm)	65 (4.73)	69.15 (4.93)	68.95 (3.82)	75.11 (5.39)	79.31 (5.89)	79 (4.51)	85 (4.85)	87.88 (5.91)	92.25 (6.24)	94.25 (5.48)
WHR (cm)	0.875 (0.03)	0.846 (0.03)	0.814 (0.02)	0.819 (0.04)	0.81 (0.02)	0.78 (0.04)	0.79 (0.03)	0.79 (0.02)	0.79 (0.03)	0.78 (0.04)
WHTR (%)	44.18 (2.20)	43.10 (1.72)	40.04 (2.46)	41.67 (1.62)	41.62 (2.78)	39.79 (1.85)	40.10 (2.62)	40.21 (2.30)	41.42 (2.09)	40.99 (2.82)
FAT (%)	21.62 (3.88)	21.25 (2.67)	19.60 (2.91)	20.40 (3.23)	21.63 (3.89)	18.66 (2.41)	16.29 (3.12)	15.94 (3.15)	17.08 (2.02)	16.57 (3.08)
FFM (%)	78.37 (3.88)	78.74 (2.67)	80.39 (2.91)	79.59 (3.23)	78.36 (3.89)	81.33 (2.41)	83.70 (3.12)	84.05 (3.15)	82.91 (2.02)	83.42 (3.08)
TBW (%)	57.42 (2.86)	57.69 (1.95)	58.88 (2.13)	58.25 (2.39)	57.34 (2.80)	59.57 (1.79)	61.25 (2.29)	61.56 (2.29)	60.71 (1.47)	61.03 (2.26)
BMI (kg/m^2^)	16.67 (1.70)	17.43 (1.69)	16.24 (1.67)	18.17 (1.58)	18.84 (2.29)	18.15 (1.52)	19.55 (1.80)	20.21 (2.30)	22.49 (2.14)	22.48 (1.95)
General Fitness										
The Ruffier test (points)	14.16 (2.67)	13.27 (2.74)	10.64 (4.97)	13.68 (5.23)	12.26 (4.63)	14.63 (4.98)	14.08 (3.54)	14.51 (3.09)	13.63 (2.31)	15.41 (3.36)
5 m dash (s)	1.440 (0.08)	1.354 (0.13)	1.35 (0.09)	1.32 (0.09)	1.27 (0.13)	1.22 (0.05)	1.22 (0.07)	1.218 (0.12)	1.176 (0.11)	1.14 (0.07)
Grip Strength, RH (kG)	11.1 (1.73)	14.46 (3.15)	14.45 (3.58)	22.22 (9.91)	20.87 (2.03)	24.63 (7.62)	29.3 (3.53)	32.66 (6.78)	38.83 (9.54)	38.14 (13.15)

Abbreviations: HbA1c—glycated hemoglobin; WHR—waist-to-hip ratio; WHtR—waist-to-height ratio; Sum of 4 SKF (skinfolds)—triceps, subscapular, hip, calf; FAT—fat mass; FFM—fat free mass; TBW—estimated total body water; RH—right hand.

**Table 2:** Body mass index distribution of participants according to International Obesity Task Force classifications.

BMI (Cole’s Cutoffs)	Boys’ Age Groups																				Total	
	8		9		10		11		12		13		14		15		16		17			
	(*n* = 10)		(*n* = 13)		(*n* = 11)		(*n* = 9)		(*n* = 8)		(*n* = 11)		(*n* = 10)		(*n* = 9)		(*n* = 6)		(*n* = 7)		(*n* = 94)	
	*n*	%	*n*	%	*n*	%	*n*	%	*n*	%	*n*	%	*n*	%	*n*	%	*n*	%	*n*	%	*n*	%
Thin					1	1.06					1	1.06	1	1.06	1	1.06					4	4.26
Normal weight	8	8.51	11	11.70	9	9.52	9	9.57	7	7.45	10	10.64	9	9.57	7	7.45	4	4.26	6	6.38	80	85.11
Overweight	2	2.13	2	2.13	1	1.00			1	1.06					1	1.06	2	2.13	1	1.06	10	10.64
Obese																						
Total (%)		10.64		13.83		11.70		9.57		8.51		11.70		10.64		9.57		6.38		7.45		100.0
Thinness																						
Mild thinness					1	25.0															1	25.0
Moderate thinness											1	25.0	1	25.0	1	25.0					3	75.0
Severe thinness																						

Based on Cole et al. (2000, 2007).

**Table 3:** Scores of Polish boys aged 8–17 years with type 1 diabetes for 5 m dash (s; M (SD)) and handgrip strength (kg; M (SD)) in comparison with the norms for the healthy population.

Age Groups		8	9	10	11	12	13	14	15	16	17
5m dash (s)											
Type 1 Diabetes boys		1.44 (0.08)	1.35 (0.13)	1.35 (0.10)	1.32 (0.10)	1.27 (0.13)	1.22 (0.05)	1.22 (0.07)	1.21 (0.12)	1.18 (0.11)	1.14 (0.07)
norm ^†^	♦		1.35–1.40		1.20–1.25		1.08–1.12		1.04–1.08		1.00–1.04
	♦♦		1.30–1.34		1.15–1.19		1.05–1.07		1.00–1.03		0.95–0.99
	♦♦♦		<1.25		<1.14		<1.05		≤1.00		≤0.95
Above any norm (%)			46	18	78	50	100	100	89	83	100
Handgrip strength (kg)											
T1D groups		11.1 (1.72)	14.6 (3.15)	14.5 (3.58)	22.2 (9.91)	20.9 (2.03)	24.6 (7.61)	29.3 (3.53)	32.7 (6.78)	38.8 (9.54)	38.1 (13.56)
Population ^††^		10–15	12–18	14–20	17–23	20–27	23–34	28–40	33–46	39–51	42–55
Below population range (%)		10	15	36	22	0	45	20	44	33	29

♦—average; ♦♦—good; ♦♦♦—very good; ^†^—norm for youth soccer players (Zając, Waśkiewicz 1998); ^††^—population range (Dobosz, 2012).

## Data Availability

The data presented in this study are available on request from the corresponding author.
